# Breathomics: A Non-Invasive Approach for the Diagnosis of Breast Cancer

**DOI:** 10.3390/bioengineering12040411

**Published:** 2025-04-12

**Authors:** Hélène Yockell-Lelièvre, Romy Philip, Palash Kaushik, Ashok Prabhu Masilamani, Sarkis H. Meterissian

**Affiliations:** 1 Noze, 4920 Pl. Olivia, Saint-Laurent, QC H4R 2Z8, Canada; pkaushik@noze.ca (P.K.);; 2 Department of Surgery and Oncology, McGill University, Montreal, QC H4A 3J1, Canada; romy.philip@mail.mcgill.ca (R.P.); sarkis.meterissian@mcgill.ca (S.H.M.)

**Keywords:** breathomics, breath analysis, breast cancer screening, breast cancer diagnostics, electronic nose, volatile organic compounds

## Abstract

Breast cancer is the most commonly diagnosed cancer worldwide, underscoring the critical need for effective early detection methods to reduce mortality. Traditional detection techniques, such as mammography, present significant limitations, particularly in women with dense breast tissue, highlighting the need for alternative screening approaches. Breathomics, based on the analysis of Volatile Organic Compounds (VOCs) present in exhaled breath, offers a non-invasive, potentially transformative diagnostic tool. These VOCs are metabolic byproducts from various organs of the human body whose presence and varying concentrations in breath are reflective of different health conditions. This review explores the potential of breathomics, highlighting its promise as a rapid, cost-effective screening approach for breast cancer, facilitated through the integration of portable solutions like electronic noses (e-noses). Key considerations for clinical translation—including patient selection, environmental confounders, and different breath collection methods—will be examined in terms of how each of them affects the breath profile. However, there are also challenges such as patient variability in VOC signatures, and the need for standardization in breath sampling protocols. Future research should prioritize standardizing sampling and analytical procedures and validating their clinical utility through large-scale clinical trials.

## 1. Introduction

Breast cancer represents a significant global health concern, as it is currently the most commonly diagnosed cancer worldwide [1]. accounting for one third of cancer diagnoses in women [2]. With its incidence on the rise as the population ages, effective means of early detection are paramount in ameliorating interventions and decreasing cancer mortality. The current diagnostic paradigm for breast cancer typically begins with mammography as an initial screening tool. When abnormalities are detected, further investigations are conducted, including higher-resolution X-rays, ultrasound examinations, Magnetic Resonance Imaging (MRI), and, ultimately, biopsy.

These conventional diagnostic procedures present several challenges. They are often time-consuming, costly, and require the expertise of specially trained medical professionals; factors which collectively limit their accessibility. Also, the invasive nature of mammography as a screening tool can cause discomfort, pain, and anxiety in patients, enough so to represent a significant motivator for avoiding screening altogether [3,4].

Furthermore, mammography, while being one of the most effective tools for the early detection of breast cancer, presents serious limitations, particularly in women with dense breast tissue. About 40% of women have heterogeneously dense breasts, and 10% have extremely dense breasts, where the breast is almost entirely composed of fibroglandular tissue, thus potentially obscuring cancers [5,6]. The sensitivity of mammography is therefore significantly reduced, particularly for younger patients [6].

Given these limitations, there is a pressing need for alternative diagnostic approaches that are reliable, cost-effective, and less invasive. Such methods could potentially improve early detection rates, reduce healthcare costs, and increase accessibility to breast cancer screening, particularly in resource-limited settings. It is within this context that breathomics emerges as a promising avenue for breast cancer diagnosis, offering the potential for quick, easy, and non-invasive screening.

## 2. Breathomics as a Non-Invasive Diagnostic Tool for Breast Cancer Detection

**Figure 1:**
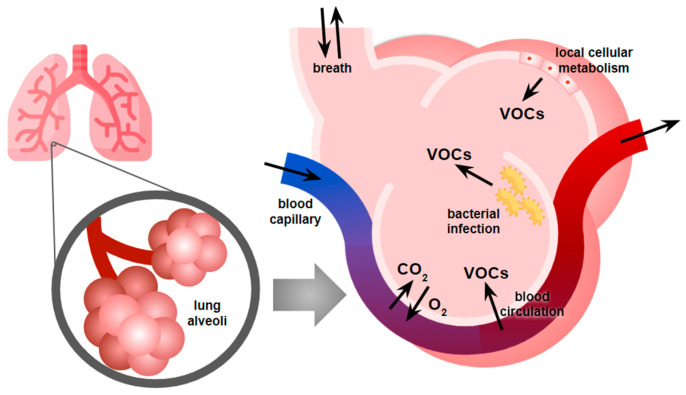
Endogenous sources for VOCs found in human breath.

Breathomics constitutes the comprehensive analysis of exhaled breath to identify and measure Volatile Organic Compounds (VOCs) that may serve as biomarkers for various physiological and pathological conditions. The human breath contains hundreds of VOCs that originate from normal metabolic processes, pathological conditions, environmental exposure, or often many sources at once. These compounds can provide valuable information about an individual’s health status [7,8]. Figure 1 illustrates the multiple sources of breath VOCs: many originate from blood, permeating the lung’s alveoli, while some originate locally from the metabolism of lung cells. Characteristic ratios of different VOCs in the breath can distinguish between healthy and pathological states. For example, in the case of respiratory infections, pathogens emit specific VOCs that would not otherwise be present in exhaled breath, allowing their identification [9]. The human body also absorbs external VOCs, and this intake, like the emissions, can vary depending on the overall health of the individual. All these factors contribute to creating a complex breath signature that is reflective of an individual’s overall health status.

Over the last decade, breathomics has emerged as a potential diagnostic tool, with research directed at its application to breast cancer diagnosis in particular [10]. The endogenous metabolic origin of breath VOCs associated with breast cancer primarily involves alterations in cellular metabolism due to cell activity. These metabolic changes lead to the alteration of the emission rate of VOCs, or to the production of specific VOCs. Cancer cells located anywhere in the body are in close proximity to blood vessels; therefore, VOCs from these cells can travel from the blood to the breath through the lungs.

Bioassays that are commonly used to diagnose different subtypes of breast cancers target specific biomarkers, mostly proteins such as hormone receptors (Human Epidermal Growth Factor Receptor 2 (HER2), Estrogen Receptor (ER), Progesterone Receptor (PR)). These bioassays function with the lock-and-key model, where one test targets one single molecule. Breath VOCs are typically small molecules that are the final product of a complex cascade of biochemical processes. Since the individual breath VOCs can be the product of many different biological origins, they can hardly be targeted as single, specific biomarkers for a given health condition. What constitutes a biomarker in breathomics is the collective response of the body to a certain condition, which typically leads to a collective change in the concentrations of several VOCs in exhaled breath. This can lead to highly specific results for breath tests, since the collective response of dozens of individual biomarkers provides a disease-specific signature. While the field is very actively researched, the specific biological mechanisms that generate volatile metabolites remain under investigation.

Several studies have identified VOCs in exhaled breath that are linked to breast cancer. These VOCs originate from altered metabolic pathways due to tumor activity, oxidative stress, and inflammation. Cancer cells are known to rely heavily on lipids, in particular, fatty acids, for anabolic and catabolic needs [11]. Therefore, most of the identified breath VOCs associated with the presence of breast cancer are attributed to lipid peroxidation from oxidative stress: mostly alkanes (heptane [12]) and aldehydes (hexanal [12,13], heptanal [13,14]), but also ketones (cyclohexanone [15,16]) and alcohols (2-ethylhexanol [12,15,17]). While many VOCs are common among various cancer types, research has shown that the overall VOC pattern is distinctive enough to differentiate not only between the breath profiles of patients with different cancers [15,18] but also among breast cancer patients with varying subtypes [12,15].

## 3. Technologies Used in Breathomics Analysis

From research performed during the last decade, breathomics established that it can identify biomarkers for various diseases, including cancers, respiratory disorders, and metabolic conditions, allowing for early detection and intervention [19,20]. The identification of specific VOCs in exhaled breath is central to establishing breath-based cancer diagnostics, and, to date, a lot of work has been conducted in an attempt to identify individual breath biomarkers specifically associated with breast cancer.

**Figure 2:**
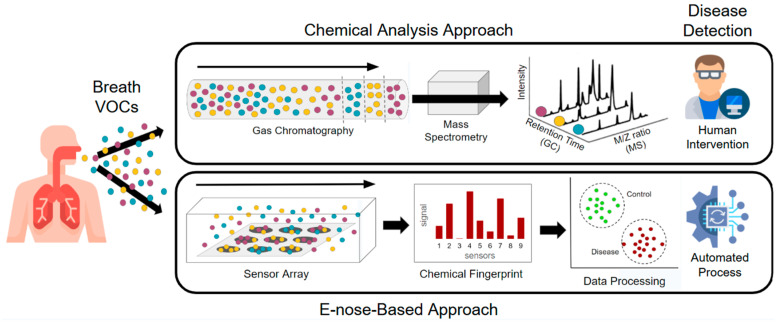
An illustration of the two main approaches currently used in breathomics. While fundamental research identifying individual biomarkers is conducted on complex, lab-confined instrumentation, new portable tools like e-nose focus on quick generation of breath patterns, involving the collective contribution of all breath VOCs.

Currently, most research is conducted using intensive, lab-based instrumentation like Gas Chromatography coupled to Mass Spectrometry (GC-MS), allowing the identification and potential quantification of breath biomarkers (Figure 2). GC-MS is renowned for its ability to separate and identify a vast array of VOCs present in exhaled breath down to trace levels, with capacity that is unmatched by other analytical techniques. Since the concentration of VOCs in the breath is still relatively low (in the ppb range), GC-MS requires sample preconcentration, often using sorbent tubes and Thermal Desorption (TD), or Solid Phase Microextraction (SPME). Using GC-MS, a few research teams have already tentatively identified a few breath volatile biomarkers associated with breast cancer, which can reveal subtle changes in metabolic pathways associated with cancer. For instance, the research team of Michael Phillips, a pioneer in the field, has used TD-GC-MS early on to identify a series of methylated alkanes associated with oxidative stress, a complex series of biochemical processes that produces a characteristic blend of VOCs depending on the specific health condition it is linked with, such as lung cancer [21], tuberculosis [22], or aging [23].

While this fundamental research is crucial for advancing the field, there is growing interest in developing simpler, more accessible breath analysis tools for clinical use. The most popular candidate is the electronic nose (e-nose) [24,25,26,27]. They are portable, advanced devices designed to detect and identify complex mixtures of VOCs, mimicking the human sense of smell. At the heart of these devices is a sensor array that consists of multiple sensors, each engineered to respond to a broad spectrum of chemical compounds. These sensors are designed with different sensitivities, meaning they react to different aspects (chemical functions) of the VOCs present in breath. When a VOC mixture comes into contact with the sensor array, each sensor generates a distinct signal. The nature of this signal depends on the specific VOCs present, the concentration of each compound, and the interaction between the VOCs and the sensor’s active material. The diversity within the sensor array is key to its functionality, as it allows the device to capture a wide variety of chemical signals. Its function mimics the human olfactory system, which comprises only about 300 sensory receptors but can differentiate over 10,000 different scents.

The sensors composing the arrays in e-noses can be chemiresistive [28], optical (colorimetric and/or fluorometric) [29], Quartz Crystal Microbalance (QCM) [30], or Surface Acoustic Wave (SAW) [31] sensors. The most popular type of sensor is the chemiresistive sensor, a widely used type of gas sensor that detects VOCs by measuring changes in electrical resistance when gas molecules interact with the sensor’s active material. Many different materials can be used in chemiresistive sensor arrays: Metal Oxide Semiconductors (MOS) [32,33]; conducting polymers [34]; carbon-based materials such as Carbon Nanotubes (CNTs) [35] or graphene [36]; Self-Assembled Monolayer (SAM)-protected gold nanoparticles (AuNPs) [37]; and hybrid nanomaterials (ex: combination of polymer and Carbon Black (CB) [38], or MOS and CNTs [39]). The use of hybrid materials allows for further customization of each sensor to target specific chemical families of VOCs.

The most powerful aspect of a sensor array in an e-nose is its ability to generate a unique “fingerprint” or signature for each VOC mixture (Figure 2). This fingerprint is a composite of the individual sensor responses, forming a multi-dimensional data set that can be analyzed to identify a specific VOC mixture or an aroma profile. Once the sensor array generates the signal pattern, the data are processed using sophisticated pattern recognition algorithms. The e-nose must be trained with known samples to calibrate the sensor array. During training, the device learns to associate specific signal patterns with known VOC profiles. Once calibrated, the e-nose can recognize these patterns in new, unknown samples, effectively identifying different complex states and conditions.

Many preliminary research studies have already been performed using these promising devices for diagnostics purposes on diseases such as breast cancer [40], lung cancer [41], as well as Alzheimer’s and Parkinson’s disease [42]. Through the use of compact tools like e-noses, portable breath analyzers could enable on-the-spot diagnosis/screening in various settings, including clinics, homes, or remote areas with limited healthcare access. Using e-nose solutions, breathomics could potentially allow patients with chronic conditions to monitor their health status regularly without the need for frequent hospital visits or invasive procedures. It could also help tailor treatments to individual patients by providing real-time information on metabolic processes and drug responses. Furthermore, because of the relatively low cost and rapid results it offers, breathomics represents a cost-effective method for large-scale health screenings and early disease detection programs.

## 4. Research Clinical Studies on Breathomics for Breast Cancer

About two dozen breathomics clinical studies have been conducted on women with various molecular subtypes and stages of breast cancer. The key highlights of these clinical studies are summarized below in Table 1. While these studies show great variability in terms of how they were conducted, some consistent trends can be extrapolated from the results. These variations and trends are discussed further in the upcoming sections.

### 4.1. Patient Selection and Study Design

From Table 1, it can be seen that the size of the participant cohorts vary substantially across the studies, from a very small size (3 BC patients vs. 3 healthy controls, in the earliest study from Ebeler et al. [10]) to larger cohort sizes in later studies (Zhang et al. [57] tested 937 BC patients vs. 1044 healthy controls). Overall, the average total cohort size in these studies ranges between 150 and 200 participants including both breast cancer patients and healthy control populations. The studies also predominantly focused on women with histologically confirmed breast cancer, ensuring robust validation of the presence of VOC biomarkers in exhaled breath. Most studies included breast cancer patients with varying disease stages, often biopsy-proven, before the initiation of any treatment [16,46,49]. Healthy controls were carefully selected, often age-matched, with no history of breast cancer or related malignancies. For example, Phillips et al. [14] included 51 women with breast cancer, 50 with abnormal mammograms but no malignancy, and 42 healthy controls. The cohort of Barash et al. [12] included 169 patients with malignancy, 25 patients with Ductal Carcinoma In Situ (DCIS), 52 patients with benign breast conditions, and 30 healthy controls, which allowed for the assessment of the diagnostic specificity of breath VOCs in distinguishing malignant from benign breast conditions, as well as their ability to differentiate breast cancer from healthy individuals.

The mean ages of participants were typically in the 50–60 range, reflecting the demographic most at risk for breast cancer. For instance, Wang et al. [16] included women aged 25–80, stratified by disease type. Age-matching was emphasized to reduce bias, as seen in Phillips et al. [14] and Shuster et al. [48]. This was carried out since age impacts metabolism, oxidative stress, and lung function, all of which influence the VOC generation in the organ, and subsequent exhalation in breath [23]. Older individuals may have higher levels of oxidative stress-related breath VOCs, regardless of disease status, leading to reduced specificity.

### 4.2. Confounding Factors and Exclusion Criteria

Exhaled breath VOCs and their origins have been extensively researched [58,59,60] in the recent decade where several non-disease contributing factors (or confounding factors) have been identified to affect breath volatiles. To minimize the influence of these confounding factors in a breath-based clinical study, common practices for the participants include restricting exercise, smoking, and consumption of food or drink or alcohol for 2–3 h before testing. However, the extent to which these measures enhance reliability in breathomics remains to be established. In our tabulated list of breath studies on breast cancer, rigorous exclusion criteria were adopted to minimize the impact of confounding factors. The most common exclusion criteria include prior cancer diagnoses (except non-melanoma skin cancer) [12,13,14,15,16,43,44,51,52,57], acute pulmonary or systemic infections such as influenza, pneumonia or tuberculosis [12,15,16,51,52,56,57], and chronic respiratory diseases such as asthma and Chronic Obstructive Pulmonary Disease (COPD) [12,15,16,52]. In two cases, pregnant or breastfeeding women were excluded [15,16], and in one study, those on medications whose metabolism could influence VOC levels were excluded [16]. A few of the studies also excluded participants with other active malignancies or significant systemic conditions such as diabetes [53] or kidney or liver dysfunction [57], which are also known to impact the breath volatilome [61,62]. In almost all studies, breast cancer patients were required not to have undergone any treatment prior to the study in order to participate. The recent use of anesthesia [51,56] was grounds for exclusion in two studies, as they were suspected to introduce variability in breath VOC profiles. Other factors such as diet and smoking habits are known to have an impact over the breath volatiles. For example, individuals following a low carb, ketogenic diet have been demonstrated to produce higher levels of acetone on their breath, reflecting increased fat breakdown and ketone body production [63]. Smoking can introduce additional hydrocarbons, aldehydes, and other compounds into the breath [64], complicating the identification of cancer-specific biomarkers. However, only two studies excluded smokers from their cohorts [13,53].

While a lot of confounding factors have been identified and applied as exclusion criteria for the studies, their direct impact on the actual results is seldom explored. In a rare example, Yang et al. first tested an e-nose for the detection of breast cancer, excluding male patients, those with a history of asthma, diabetes mellitus, smoking, or having received chemotherapy in a vast study including 661 participants [53]. Under these conditions, they obtained a sensitivity of 86% and a specificity of 97%. However, upon the introduction of patients with comorbidities making up the exclusion criteria, they noted marked decreases in diagnostic accuracy. With their Diagnostic Odds Ratio (DOR) originally near 11, the inclusion of those with diabetes pushed the DOR to 8.51. Another study conducted by Peng et al. studied specifically the impact of different confounding factors on the performances of their e-nose device, using a separate group of 59 healthy subjects carefully divided into different categories according to gender, age, ethnicity, family history of cancer, medication, diet, or smoking habits [18]. Their e-nose analysis could not differentiate between individuals belonging to either of the different categories, demonstrating that none of these confounding factors affected their e-nose output. However, their e-nose could easily separate between the healthy controls and the patients with breast cancer. Furthermore, their study also included patients with lung cancer, colorectal cancer, and prostate cancer, and the results showed a clear separation between each group, with the prostate cancer group being less defined [18].

Several studies opted to control their patient characteristics, though no actual consensus regarding exclusion or inclusion criteria was found. While many studies stipulated that participants could not have a history of any prior malignancy or have received cancer treatments such as chemotherapy, radiation, or surgery as those factors are likely to affect the VOC profile, there were numerous studies not excluding any comorbidities, and requiring only a clear mammogram within the past year as a measure of health for their healthy control group.

### 4.3. Breath Sample Collection and Environment

The volume of breath sampled varied from 0.5 L to 2 L, with most studies sampling 1L of alveolar breath. In nearly all of the studies, the exhaled breath was first sampled and stored either in a breath bag or in a TD tube containing a sorbent material. The stored breath was then introduced into the analytical equipment or the e-nose. Typically, these breath sampling procedures have been derived from standard research protocols when using lab-based analytical methods such as GC-MS, but Point-Of-Care (POC) devices based on e-nose technology should ideally be able to sample the breath directly from the patient. However, of the six reported studies [12,18,48,50,52,53] that used an e-nose device, only one [50] used direct breathing into the device.

Alveolar breath constitutes the end tidal volume, where carbon dioxide (CO_2_) is exchanged for oxygen. It is the most relevant fraction of the whole breath, given that it is where endogenous VOCs are found in the greatest concentration [65]. The remaining, “dead space” portion of the exhaled breath, comprising air from the mouth, throat, and large airways, contains compounds influenced by environmental contaminants, residual air, and non-diagnostic sources such as oral and nasal microbiota. Including this air can dilute or obscure the VOCs of interest, reducing the sensitivity and specificity of the analysis, therefore all studies except two [10,44] opted to only sample the alveolar part of the breath. However, the methodology to precisely collect the alveolar breath varies across studies. Some sampling is more manual in nature, with dead space being collected into one breath bag, and alveolar breath into a separate one [12,14,15,16,18,43,45,47,48,49,51], whereas other methods are more automated such as capnography [53,56]. Capnography uses a Non-Dispersive Infrared (NDIR) sensor to continuously measure the level of CO_2_ in the exhaled breath. Since the alveolar breath is known to contain around 4–5% of CO_2_, capnography is able to mark the precise moment when the alveolar portion of the breath starts, and then automatically activate sampling. In one study [53], the team decided to perform breath sampling while the patients were undergoing anesthesia (Sevoflurane 2%), with the alveolar sampling being performed by the anesthesiologist by directly accessing the patient’s endotracheal intubation prior to their breast tumor resection surgery. The healthy controls from this study were sampled using the exact same procedure in order to validate their results, but this sampling method is not a practical approach that could be more conveniently used in a doctor’s office.

Nevertheless, exogenous VOCs are expected to appear in breath analysis, and research teams have used multiple approaches in order to mitigate their contribution. The most common is to sample the ambient air in the sample collection room to determine what constitutes “background” and what is endogenous. Another technique, though often found in tandem, is to control environmental and nutritional factors [12,13,18,45,46,56,65,66,67]. This has been conducted by having patients fast or refrain from certain activities (e.g., alcohol and tobacco consumption, practicing oral hygiene, etc.) for a given amount of time prior to sampling [12,14,15,45,52,56,57]. In other studies, patients have been asked to rinse their mouths with distilled water and/or breathe in clean air through filtered mouthpieces for several minutes as a form of “lung wash out” [12,18,46,56]. Though in comparing several collection methods, Di Gilio et al. found that ambient air sampling may imbue more benefit than pulmonary wash out [66]. They remark, as others have, that wash out may not actually remove environmental contaminants from exhaled breath as each VOC has specific kinetic properties that would affect its absorption, metabolism, and half-life, thereby determining the rate at which it is retained or released by the body. For example, longitudinal exposure to a pollutant could lead to certain lipophilic VOCs being taken into adipose tissue (for example, benzene-derivatives from car exhaust) They are later released into the blood, and subsequently in the breath at a slow constant rate, limiting the effectiveness of a wash out [66,68].

### 4.4. Breast Cancer Diagnosis: Sensitivity, Specificity, and Data Processing Methods

Sensitivity and specificity are two measures used to assess the accuracy of a diagnostic test. Sensitivity (true positive rate) refers to the test’s ability to correctly detect disease in individuals who actually have the disease, while specificity (true negative rate) refers to the test’s ability to correctly identify individuals who do not have the disease. While there are no absolute universal threshold values for sensitivity and specificity in clinical screening tests, the recommended values are generally expected to be around 80–90% for sensitivity and around 90–95% for specificity. For breast cancer screening, false positive rates represent invasive follow-ups, therefore higher specificity is desirable. When using mammography as a screening tool, a 2024 study demonstrated that women who received a false positive result were significantly less willing to return for a subsequent routine screening [69].

The performance metrics obtained by each breathomics study for breast cancer diagnosis can be viewed in Table 1. Overall, sensitivity values range from 60.8% [43] to 100% [52], with most values found in the 80–85% range. Values for specificity range from 40% [49] to 100% [50,52], with most values found in the 75–80% range. As can be expected, the sensitivity and specificity values reported across the studies are heavily influenced by experimental protocols, participant selection, sample handling, analytical devices, and the statistical models employed. When it comes to breath sampling, studies that employed standardized alveolar breath collection methods typically achieved better results, as the alveolar portion represents the rich metabolic VOC biomarkers released from various organs across the body. Peng et al. [18] directly compared the performances of GC-MS against their e-nose sensor array on the same set of volunteers, with the e-nose showing both higher sensitivity (90% vs. 85%) and specificity (85% vs. 75%) when discerning between breast cancer patients and healthy controls.

As shown in Table 1, except for a few studies [10,13,44,49], all the other studies have used some form of Machine Learning (ML)-based data processing to generate the sensitivity and specificity metrics. Some of these studies [15,16,46,51] were aimed at uncovering VOC biomarkers for breast cancer and the ML models built represent the best correlated VOCs from the GC-MS analysis. Most of the studies have applied linear dimensionality reduction techniques like PCA and some form of discriminant analysis technique like LDA, QDA, CDA, PLSDA, and OPLSDA [70,71]. Some groups used a linear SVM [45,47,48,53,55] classifier. The study from Diaz de Leon-Martinez et al. [52] applied RBF-SVM (non-linear) along with a linear CDA method for analysis, which likely suffered from an overfit. To overcome statistical overfit in the analysis, many studies use the standard train/validation/test split technique [70]. For example, Liu et al. [56] used three splits of their data set grouped as training (50%), validation (20%), and test (30%) groups and applied several combinations of these folds to perform cross validation with their Random Forest Classifier. Zhang et al. [57] also used a very similar approach. The best method for cross validation however is Leave One Out Cross Validation (LOOCV) [70], which was reported as the applied method in a few studies [12,43,55] while the details were not elaborated. However, when it comes to best practice in ML method applications, studies from Lee et al. [45] and Patterson et al. [47] conducted thorough work of applying both linear (PCA) and non-linear (Laplacian Eigenmaps) for the dimensionality reduction step and subsequently trained LDA, QDA, and SVM classifiers on both approaches.

While the goal of most studies was to establish different volatile breath profiles that differentiate breast cancer patients from healthy individuals and patients with benign breast tumors, very few tried to investigate whether breath profiles could differentiate between patients with different types of breast cancer. The only one, to our knowledge, is the one conducted by Barash et al. [12] to examine differences in breath VOCs associated with the molecular subtype of breast cancer, using both GC-MS and e-nose. Their study explores the metabolic impact of specific mutations within cancer cells, resulting in breast cancer subtypes such as Luminal 1, Luminal 2, HER2+, and triple negative, which can result in altered VOC profiles. Distinct VOC patterns associated with different cancer subtypes were observed, suggesting that specific metabolic pathways influenced by genetic mutations contribute to the volatile signature. These mutations drive changes in metabolic pathways (e.g., lipid peroxidation, oxidative stress), producing unique VOC patterns reflective of the tumor’s genetic background. They found that each subtype could be discerned with accuracies of 81–88% for Luminal A, 78–86% for Luminal B, and 83–90% for triple negative cancer. For each of these, the sensitivity was approximately 80%. Additionally, HER2 status was identified independent of hormone receptor status, and interestingly, when HER2 was equivocal, it was found to be HER2+ 35% of the time during the VOC analysis, much like with reported Fluorescence In Situ Hybridization (FISH) data.

## 5. Advantages and Limitations of the Approach, Future Prospects, and Directions

**Figure 3:**
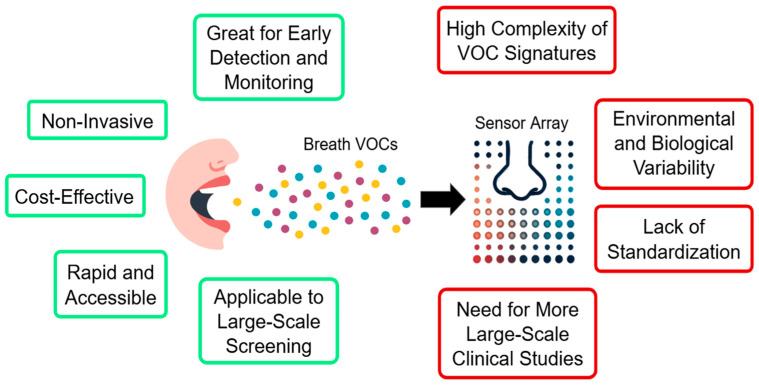
A schematization of the main advantages and challenges associated with the use of an e-nose for disease diagnosis using breath.

One of the most significant advantages of breathomics in breast cancer screening is its non-invasive nature. Breath analysis involves collecting exhaled air, which is far more comfortable and less risky for patients compared to traditional screening methods such as biopsies, mammograms, or blood tests. This could lead to increased patient compliance, especially in populations that are hesitant to undergo more invasive procedures. Breath analysis can potentially provide rapid results, especially when advanced technologies such as e-noses are employed (Figure 3). In an e-nose-based breathomics approach, there is no need to capture, store, and analyze the breath samples off-line, as the sampling and AI-based interpretation is performed in real time within the device. For this reason, the cost per screening test in the case of e-nose breathomics only includes the consumables like the mouthpiece and the AI model compute cost. This makes the e-nose approach a convenient and a low-cost option for early screening, especially in resource-limited settings where access to complex imaging technology may be scarce. Breathomics also has the potential to enable the detection of breast cancer at earlier stages through the identification of breath VOC mixture patterns associated with micro-calcifications and benign lesions, which are potential breast cancer precursors [48].

However, because breathomics is still an emerging field, its applicability to the diagnosis of breast cancer needs further validation and development, since having a high sensitivity and low false positive result rate (high specificity) is imperative. From the analysis in Section 4, it is clear that across multiple clinical studies with different breath sampling devices, the exhaled breath from breast cancer patients seems to contain a unique and complex mixture of VOCs that can be clearly separated from the control population’s breath samples. However, the complex VOC mixture can present variations based on a number of factors such as the cancer subtype [12], development stage [57], and the patient’s overall health [53]. In women more specifically, factors that have a play on the circulating hormonal levels such as the menstrual cycle [72], the use of oral contraceptives [72,73], and age (pre-or post-menopause) [73] have demonstrated an impact on the breath volatilome. Finally, breath VOCs can be influenced by external factors such as environmental pollutants, diet, and smoking habits [63,74]. This variability needs to be taken into account to ensure the accuracy of results and generalizability of breathomics as a screening tool for breast cancer.

On the detection technology side, the spectrometry instruments currently being used lack portability and affordability and require time-consuming data processing for identifying biomarkers from the spectrum of generated data. For e-noses, recent breakthroughs have enabled mass production, allowing both portability and affordability. It is also observed that e-nose devices are generally better performing as a breathomics tool for breast cancer in the detection metrics in comparison to spectrometry-based devices. However, so far, all e-nose-based studies suffer from non-standard breath sampling techniques and different mechanisms to capture the alveolar breath. Given the promise of e-nose technologies to be a low cost, real-time, POC breathomics solution, it is imperative that they integrate a standardized direct breath sampling technique and capnography logic to eliminate any variability from external factors and exogenous VOCs. E-nose devices are also leveraging advanced AI algorithms which are trained on large amounts of data, enabling them to handle confounders and help make more effective diagnostic decisions. ML methods like transfer learning can also help effectively compensate for common issues in e-nose sensors such as sensor drift [70,71,75] and humidity sensitivity [76].

On the clinical side, large-scale clinical trials are necessary to validate the generalizability of breathomics in detecting breast cancer reliably across diverse populations and further address the impact of confounding factors. Rigorously designed clinical studies, that take into account both the endogenous and exogenous factors that affect breath VOCs and adapt their sampling and analysis methods accordingly, can provide critical improvements in breast cancer breathomics.

On a comparative note, there are other novel methods like liquid biopsy [77] and AI-assisted mammography [78] that are being developed for breast cancer screening. In liquid biopsy, the most effective method is to track Circulating Tumor DNA (ctDNA) in patient blood samples. However, it does require a blood draw and the latest results [77] indicate that ctDNA technology could be used for uniquely identifying the recurrence of breast cancer. As for AI-assisted mammography, despite the reported performance enhancement [78], the invasive aspect of mammography is still present.

## 6. Conclusions

Breathomics is a promising, non-invasive approach to breast cancer screening that has the potential to offer rapid, cost-effective, and accessible screening for large populations. While breathomics shows great promise, it is important to note that the field is still developing. Many studies have been conducted, but the results have great variability due to techniques used for breath sampling and analysis. Future challenges include standardizing collection and analysis methods, developing detection technologies that are both sensitive and specific, and validating results through large-scale clinical trials in order to limit the effect of confounding factors. As research progresses, breathomics has the potential to revolutionize diagnostics by providing quick, easy, and non-invasive health assessments. Future research is likely to focus on refining the detection methods and integrating breathomics with other screening tools to maximize its utility in clinical practice. There is also a need to clearly and repeatedly identify breath biomarkers associated with breast cancer. Then, a more in-depth analysis addressing the metabolic origins of the observed breath VOCs associated with breast cancer will help to fully understand the complex relationship between the disease and breath.

## Figures and Tables

**Table 1:** Overview of different studies on breathomics for breast cancer diagnosis.

Top Author, Year, Ref.	Study Population	Equipment and Data Processing Methods	Sensitivity	Specificity
Ebeler, 1997, [10]	3 BC patients/3 healthy controls	GC-FPD		
Phillips, 2003, [43]	51 BC patients/50 women with abnormal mammogram but no cancer/42 healthy controls	TD-GC-MS with FSDA (SPSS software version 8.0)	Model 1: 88.2% Model 2: 60.8%	Model 1: 73.8% Model 2: 82.0%
Phillips, 2006, [14]	51 BC patients/42 healthy controls	TD-GC-MS with Fuzzy Logic	93.80%	84.60%
McCulloch, 2006, [44]	31 BC patients/55 lung cancer patients/83 healthy controls	trained dog	88%	98%
Lee, 2009, [45]	17 BC patients/24 healthy controls	TD-GC-MS with Laplacian Eignemaps and LDA, QDA, SVM	75%	75%
Peng, 2020, [18]	18 BC patients/30 lung cancer patients/26 colon cancer patients/18 prostate cancer/82 healthy controls	NaNose e-nose and GC-MS with PCA	90%	85%
Phillips, 2010, [46]	54 BC patients/204 healthy controls	TD-GC-MS with Weighted Digital Analysis	75.3%	84.8%
Patterson, 2011, [47]	20 BC patients/20 healthy controls	TD-GC-MS with Laplacian Eignemaps and LDA, QDA, SVM	72%	64%
Shuster, 2011, [48]	13 BC patients/16 patients with benign breast tumor/7 healthy controls	NaNose e-nose with PCA and SVM	94%	80%
Mangler, 2012, [49]	10 BC patients/10 healthy controls	TD-GC-MS with SPSS version 15.0 analysis	80 to 100%	40 to 70%
Wang, 2014, [16]	85 BC patients/45 healthy controls	SPME-GC-MS with PCA and PLSDA, OPLSDA		
Li, 2014, [13]	22 BC patients/17 breast benign tumors/24 healthy controls	GC-MS with Fisher Discriminant Analysis using SPSS	72.7%	91.7%
Barash, 2015, [12]	169 malignant BC patients/25 DCIS/52 benign breast conditions/30 controls	GC-MS and NaNose e-nose with DFA	70 to 88%	71 to 87%
Herman-Saffar, 2018, [50]	48 BC patients/45 healthy controls	MK4 and Cyranose e-noses with ANN	MK4: 89 to 93% Cyranose: 88 to 92%	MK4: 95 to 100% Cyranose: 78 to 85%
Phillips, 2018, [51]	54 BC patients/124 healthy controls	GC-MS and GC-SAW with Weighted Digital Analysis	85%	85%
Díaz de León-Martínez, 2020, [52]	262 BC patients/181 healthy controls	Cyranose 320 e-nose with CDA and RBF-SVM	100%	100%
Zhang, 2020, [15]	71 BC patients/54 gastric cancer patients/78 healthy controls	HS-GC-MS with PCA and PLSDA	93.59%	71.62%
Yang, 2021, [53]	351 malignant BC/88 healthy controls/222 benign breast tumors	Cyranose 320 e-nose with different ML models	86%	97%
Nakayama, 2022, [54]	45 BC patients/51 healthy controls	SIFT-MS with PCA and MLR	86.3%	55.6%
Naz, 2022, [55]	71 BC patients/40 healthy controls	TD-IR-CRDS with SVM	86.8%	75.0%
Liu, 2023, [56]	465 BC patients/4504 healthy controls	HPPI-TOFMS with RF	89.16%	87.70%
Zhang, 2024, [57]	937 BC patients/1044 healthy controls	HPPI-TOFMS with RF	85.9%	90.4%

BC: breast cancer; GC: Gas Chromatography; MS: Mass Spectrometry; TD: Thermal Desorption; FPD: Flame Photometric Detector; SPSS: Statistical Package for the Social Sciences; FL: Fuzzy Logic; PCA: Principal Component Analysis; SVM: Support Vector Machine; PLSDA: Partial Least-Squares Discriminant Analysis; OPLSDA: Orthogonal PLSDA; DFA: Deterministic Finite Automaton; ANN: Artificial Neural Network; LE: Laplacian Eigenmaps; SIFT: Selected Ion Flow Tube; MLR: Multiple Logistic Regression; HPPI-TOFMS: High-Pressure Photon Ionization Time Of Flight MS; RF: Random Forest; RBF-SVM: Radial Basis Function SVM; LDA: Linear Discriminant Analysis; QDA: Quadratic Discriminant Analysis; CDA: Canonical Discriminant Analysis.
